# Investigation of post-transcriptional gene regulatory networks associated with autism spectrum disorders by microRNA expression profiling of lymphoblastoid cell lines

**DOI:** 10.1186/gm144

**Published:** 2010-04-07

**Authors:** Tewarit Sarachana, Rulun Zhou, Guang Chen, Husseini K Manji, Valerie W Hu

**Affiliations:** 1 Department of Biochemistry and Molecular Biology, The George Washington University Medical Center, 2300 Eye St NW, Washington, DC 20037, USA; 2 Laboratory of Molecular Pathophysiology, National Institute of Mental Health, National Institutes of Health, 9000 Rockville Pike, Bethesda, MD 20892, USA

## Abstract

**Background:**

Autism spectrum disorders (ASD) are neurodevelopmental disorders characterized by abnormalities in reciprocal social interactions and language development and/or usage, and by restricted interests and repetitive behaviors. Differential gene expression of neurologically relevant genes in lymphoblastoid cell lines from monozygotic twins discordant in diagnosis or severity of autism suggested that epigenetic factors such as DNA methylation or microRNAs (miRNAs) may be involved in ASD.

**Methods:**

Global miRNA expression profiling using lymphoblasts derived from these autistic twins and unaffected sibling controls was therefore performed using high-throughput miRNA microarray analysis. Selected differentially expressed miRNAs were confirmed by quantitative reverse transcription-polymerase chain reaction (qRT-PCR) analysis, and the putative target genes of two of the confirmed miRNA were validated by knockdown and overexpression of the respective miRNAs.

**Results:**

Differentially expressed miRNAs were found to target genes highly involved in neurological functions and disorders in addition to genes involved in gastrointestinal diseases, circadian rhythm signaling, as well as steroid hormone metabolism and receptor signaling. Novel network analyses of the putative target genes that were inversely expressed relative to the relevant miRNA in these same samples further revealed an association with ASD and other co-morbid disorders, including muscle and gastrointestinal diseases, as well as with biological functions implicated in ASD, such as memory and synaptic plasticity. Putative gene targets (*ID3 *and *PLK2*) of two RT-PCR-confirmed brain-specific miRNAs (hsa-miR-29b and hsa-miR-219-5p) were validated by miRNA overexpression or knockdown assays, respectively. Comparisons of these mRNA and miRNA expression levels between discordant twins and between case-control sib pairs show an inverse relationship, further suggesting that *ID3 *and *PLK2 *are *in vivo *targets of the respective miRNA. Interestingly, the up-regulation of miR-23a and down-regulation of miR-106b in this study reflected miRNA changes previously reported in post-mortem autistic cerebellum by Abu-Elneel *et al*. in 2008. This finding validates these differentially expressed miRNAs in neurological tissue from a different cohort as well as supports the use of the lymphoblasts as a surrogate to study miRNA expression in ASD.

**Conclusions:**

Findings from this study strongly suggest that dysregulation of miRNA expression contributes to the observed alterations in gene expression and, in turn, may lead to the pathophysiological conditions underlying autism.

## Background

Autism spectrum disorders (ASD) is a collective term used to describe neurodevelopmental disorders with a pattern of qualitative abnormalities in three functional domains: reciprocal social interactions, communication, and restrictive interests and/or repetitive behaviors [1]. There is strong evidence that 10 to 15% of ASD cases may be etiologically related to known genetic disorders, such as fragile X syndrome, tuberous sclerosis complex, and Rett syndrome [2,3]. However, the etiology of ASD in most cases remains unknown, as is the explanation for the strong male:female gender bias (at least 4:1) [4]. With regard to identifying genes associated with idiopathic autism, which represents 80 to 90% of ASD cases, a number of previous studies have conducted genome-wide scans to ascertain genetic linkage to, or association with, ASD. To date, autism susceptibility loci have been identified on almost every chromosome, especially chromosomes 2q [5], 3q [6], 5p [7], 6q [8], 7q [5,9], 11p [7], 16p [5], and 17q [7,10]. No single chromosomal location, however, has been found to be highly significant, and no genetic variation or mutation within these regions has been found to account for more than 1% of ASD cases. Copy number variation has also been associated with ASD, and the most recent whole genome scan performed by The Autism Consortium (2008) revealed a recurrent microdeletion and a reciprocal microduplication on chromosome 16p11.2 [11]. Moreover, a number of publications have demonstrated the relevance of particular genes to ASD, and numerous candidate genes for autism have been identified, including *NLGN3/4 *[12,13], *SHANK3 *[14], *NRXN1 *[15], and *CNTNAP2 *(Contactin associated protein-like 2) [16-18]. Interestingly, all of these genes function at the synapse, thereby focusing attention on dysregulation of synapse formation as a neuropathological mechanism in ASD [19,20]. However, studying a single ASD candidate gene at a time is not likely to provide a comprehensive explanation of all pathophysiological conditions associated with these disorders, which are believed to result from dysregulation of multiple genes.

To examine global transcriptional changes associated with ASD, Hu and colleagues [21] examined differential gene expression with DNA microarrays using lymphoblastoid cell lines (LCLs) from discordant monozygotic twins, one co-twin of which was diagnosed with autism while the other was not. They found that a number of genes important to nervous system development and function were among the most differentially expressed genes. Furthermore, these genes could be placed in a relational gene network centered on inflammatory mediators, some of which were increased in the autopsied brain tissue of autistic patients relative to non-autistic controls (for example, IL6) [22]. Inasmuch as monozygotic twins share the same genotype, the results of this study further suggested a role for epigenetic factors in ASD.

MicroRNAs (miRNAs) as well as other factors such as DNA methylation and chromatin remodeling are thus likely candidates in the epigenetic regulation of gene expression. miRNAs are endogenous, single-stranded, non-coding RNA molecules of approximately 22 nucleotides in length that negatively and post-transcriptionally regulate gene expression. The biogenesis and suppressive mechanisms of miRNAs have been comprehensively described in many studies [23-27], and include miRNA-mediated translational repression that may also ultimately lead to degradation of the transcript. miRNAs are involved in nervous system development and function [28-31]. In addition, disrupted miRNA function has been proposed to be associated with a number of neurological diseases, such as fragile X syndrome [32-35], schizophrenia [36], and spinal muscular atrophy [37]. Recently, two studies have reported differential expression of miRNA in ASD, one using LCLs as an experimental model [38], and the other interrogating miRNA expression directly in autistic and nonautistic brain tissues [39]. However, neither of these studies demonstrated correlation between the differentially expressed miRNA and differential expression of the putative target genes or gene products.

We postulated that altered miRNA expression would result, in part, in altered expression of its target genes. Therefore, we employed miRNA microarrays to study the miRNA expression profiles of LCL from male autistic case-controls, which included monozygotic twins discordant for ASD and their nonautistic siblings as well as autistic and unaffected siblings. miRNA expression profiling revealed significantly differentially expressed miRNAs whose putative target genes are associated with neurological diseases, nervous system development and function, as well as other co-morbid disorders associated with ASD, such as gastrointestinal, muscular, and inflammatory disorders. The goal of this study was to reveal dysregulation in miRNA levels that are inversely correlated with altered levels of target genes that, in turn, may be associated with the underlying pathophysiology of ASD, and to provide a better understanding of the role of miRNAs as a post-transcriptional gene regulatory mechanism associated with ASD.

## Methods

### Experimental model and cell culture

LCL derived from peripheral lymphocytes of 14 male subjects were obtained from the Autism Genetic Resource Exchange (AGRE, Los Angeles, CA, USA). The subjects included three pairs of monozygotic twins discordant for diagnosis of autism, a normal sibling for two of the twin pairs, two pairs of autistic and unaffected siblings, and a pair of normal monozygotic twins. These cell lines had all been used previously for gene expression profiling [21,40] and thus allowed us to compare miRNA expression profiles with mRNA expression levels across the affected and control samples from both studies. The frozen cells were cultured in L-Glutamine-added RPMI 1640 (Mediatech Inc., Herndon, VA, USA) with 15% triple-0.1 μm-filtered fetal bovine serum (Atlanta Biologicals, Lawrenceville, GA, USA) and 1% penicillin-streptomycin-amphotericin (Mediatech Inc.).

According to the protocol from the Rutgers University Cell and DNA Repository (which contains the AGRE samples), cultures were split 1:2 every 3 to 4 days, and cells were harvested for miRNA isolation 3 days after a split, while the cell lines were in logarithmic growth phase. All cell lines were cultured and harvested at the same time with the same procedures and reagents to minimize the differences in miRNA expression that might occur as a result of different cell and miRNA preparations.

### miRNA isolation

LCLs were disrupted in TRIzol Reagent (Invitrogen, Carlsbad, CA, USA) and miRNAs were then extracted from the TRIzol lysate using the mirVana miRNA Isolation Kit (Ambion, Austin, TX, USA) according to the manufacturers' protocols. Briefly, ethanol (100%) was added to TRIzol-extracted, purified RNA in water to bring the samples to 25% ethanol and the mixture was then passed through the mirVana glass-fiber filter, which allowed passage of small RNA in the filtrate. Ethanol was added to the filtrate to increase the ethanol concentration to 55%, and the mixture was passed through the second glass-fiber filter, which immobilized the small RNAs. After washing, the immobilized small RNAs were eluted in DNase-RNase-free water (Invitrogen), yielding an RNA fraction highly enriched in small RNA species (≤ 200 nucleotides). The concentration of the small RNAs in the final fraction was then measured with a NanoDrop 1000 spectrophotometer (Thermo Fisher Scientific, Wilmington, DE, USA). To enable comparison of miRNA expression patterns across all of the samples, equal amounts of miRNAs from unaffected siblings and normal control individuals were pooled to make a common reference miRNA that was co-hybridized with each sample on the miRNA microarray.

### miRNA microarray analysis

Custom-printed miRNA microarrays were used to screen miRNA expression profiles of LCLs from autistic and normal or undiagnosed individuals. The array slides were printed in the Microarray CORE Facility of the National Human Genome Research Institute (NHGRI, NIH, Bethesda, MD, USA). The complete set of non-coding RNAs printed in triplicate on Corning epoxide-coated slides (Corning Inc., Corning, NY, USA) is shown in Additional file 1, with the subset of human miRNAs shown on the second sheet of the Excel workbook. Although the printed arrays also included miRNA from rat and mouse species as well as some small nucleolar RNAs, these were not considered in our analyses. miRNA labeling and microarray hybridization were performed using Ambion's miRNA Labeling Kit and Bioarray Essential Kit, respectively, according to the manufacturer's instructions. Briefly, a 20- to 50-nucleotide tail was added to the 3' end of each miRNA in the sample using *Escherichia coli *Poly (A) polymerase. The amine-modified miRNAs were then purified and coupled to amine-reactive NHS-ester CyDye fluors (Amersham Biosciences, Piscataway, NJ, USA). A reference design was used for microarray hybridization in this study. The sample miRNAs were coupled with Cy3, whereas the common reference miRNA was coupled with Cy5, and two-colored miRNA microarray analyses were carried out by co-hybridizing an equal amount of both miRNA samples onto one slide.

After hybridization and washing, the microarrays were scanned with a ScanArray 5000 fluorescence scanner (PerkinElmer, Waltham, MA, USA) and the raw pixel intensity images were analyzed using IPLab image processing software package (Scanalytics, Fairfax, VA, USA). The program performs statistical methods that have been previously described [41] to locate specific miRNAs on the array, measure local background for each of them, and subtract the respective background from the spot intensity value (average of triplicate spots). Besides the background subtraction, the IPLab program was also used for within-array normalization and data filtering. Fluorescence ratios within the array were normalized according to a ratio distribution method at confidence level = 99.00. The filtered data from the IPLab program were then uploaded into R version 2.6.1 software package to perform array normalization across all of the samples based upon quantile-quantile (Q-Q) plots, using a procedure known as quantile normalization [42]. After normalization, 1,237 miRNAs were detectable above background.

### Assessing significance of miRNA expression

To identify significantly differentially expressed miRNA, the normalized data were uploaded into the TIGR Multiexperiment Viewer (TMeV) 3.1 software package [43,44] to perform statistical analyses on the microarray data as well as cluster analyses of the differentially expressed genes. Pavlidis template matching analyses [45] were carried out to identify significantly differentially expressed probes between autistic and control groups (*P *≤ 0.05). Cluster analyses were performed with the significantly differentially expressed miRNAs using the hierarchical cluster analysis program within TMeV, based on Euclidean distance using average linkage clustering methods. Principal component analysis was further employed to reduce the dimensionality of the microarray data and display the overall separation of samples from autistic and control groups.

### Prediction of the potential target genes

The lists of the potential target genes of the differentially expressed miRNAs were generated using miRBase [46] where the miRanda algorithm is used to scan all available mRNA sequences to search for maximal local complementarity alignment between the miRNA and the 3' UTR sequences of putative predicted mRNA targets. The benefit of using this program is that it also provides *P*-orthologous-group (*P*-org) values, which represent estimated probability values of the same miRNA family binding to multiple transcripts for different species in an orthologous group. The values are calculated from the level of sequence conservation between all of the 3' UTRs according to the statistical model previously described [47]. Only target sites for which the *P*-org value was < 0.05 were included to minimize false positive predictions. The number of target genes was different for each miRNA, but the range of targets per miRNA was between 600 and 1,200 protein-coding genes.

### Preliminary functional analyses of the potential target genes

Ingenuity Pathway Analysis (IPA) version 6.0 (Ingenuity Systems, Redwood City, CA, USA) and Pathway Studio version 5 (Ariadne Genomics, Rockville, MD, USA) network prediction software were used to identify gene networks, biological functions, and canonical pathways that might be impacted by dysregulation of the differentially expressed miRNAs, using the lists of predicted target genes of each differentially expressed miRNA to interrogate the gene databases. The Fisher exact test was used to identify significant pathways and functions associated with the gene datasets.

### miRNA TaqMan qRT-PCR analysis

Among the differentially expressed miRNAs, four brain-specific or brain-related miRNAs (hsa-miR-219, hsa-miR-29, hsa-miR-139-5p, and hsa-miR-103) were selected for confirmation analysis by miRNA TaqMan quantitative reverse-transcription PCR (qRT-PCR) assays (Applied Biosystems, Foster City, CA, USA). Small nucleolar RNA, C/D box 24 (RNU24) was used as an endogenous control in all qRT-PCR experiments. According to the Applied Biosystems TaqMan MicroRNA Assay protocol, cDNA was reverse transcribed from 10 ng of total RNA using specific looped miRNA RT primers, which allow for specific RT reactions for mature miRNAs only. The cDNA was then amplified by PCR, which uses TaqMan minor groove binder probes containing a reporter dye (FAM dye) linked to the 5' end of the probe, a minor groove binder at the 3' end of the probe, and a non-fluorescence quencher at the 3' end of the probe. The design of these probes allows for more accurate measurement of reporter dye contributions than possible with conventional fluorescence quenchers.

### Meta-analysis of gene expression data for these same samples

A meta-analysis was performed to correlate differential miRNA expression with gene expression data that had previously been obtained by our laboratory using the same samples. However, because the discordant twin study [21] and that involving affected-unaffected sib pairs [40] were performed using a different experimental design for microarray hybridization (that is, direct sample comparison on the same array for the twin samples and a reference design for the sib-pair analysis that involved co-hybridization of each sibling sample with Stratagene Universal human reference RNA), the expression data from the sib-pair study was reanalyzed in order to report differences as log_2 _expression ratios between the affected and unaffected siblings, which is the expression format used in the twin study. Data filtration was performed using TMeV version 3.1 software [43] to extract only genes for which expression values were present in at least four out of seven comparisons. The filtered data were then uploaded into the R statistical software package [48] to carry out quantile normalization. After global data distribution and normalization of data to the same level to enable comparison of gene expression data across the combined set of samples, a one-class *t*-test analysis was conducted across all log_2 _ratios using TMeV, and significantly differentially expressed genes were identified as those with *P*-values < 0.05. In order to capture the largest number of putative target genes of the differentially expressed miRNAs for our correlation analysis, we performed the *t*-test without multiple sample correction. The complete list of differentially expressed genes is provided in Additional file 2.

### Correlation between the expression of the target genes and the candidate miRNAs

**Figure 1:**
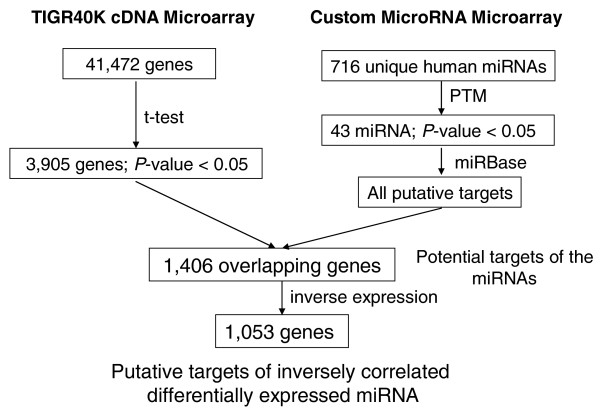
**Schematic flow diagram describing procedures used to identify inversely correlated differentially expressed putatitve target genes of the differentially expressed miRNAs**. Tens of thousands of putative target genes are associated with the 43 differentially expressed miRNAs, some of which are overlapping between different miRNAs. For the correlation analyses, we used all of the putative target genes.

To identify the differentially expressed genes potentially regulated by the differentially expressed miRNAs in autistic individuals, the overlapping genes between the significant gene list from the one-class *t*-test (*P *< 0.05) and the list of the potential target genes of all the differentially regulated miRNAs were identified. Figure 1 shows a schematic of the procedure used to correlate miRNA and putative target genes. To correlate miRNA expression with putative target gene expression, the average log_2 _expression ratios of miRNA for autistic versus unaffected groups were calculated and then compared against the average log_2 _mRNA expression ratios for these same groups. Only the target genes that were expressed in the opposite direction from that of the pertinent miRNAs were extracted for functional analyses. Although miRNA often acts as a translational repressor in mammalian cells, the targeted mRNA species is often delivered to P-bodies, where it is eventually degraded [49]. Thus, we decided to perform pathway analyses only on those genes whose mRNA changes were directionally opposite to the change in miRNA expression, while acknowledging that other mRNA species may also be potential targets of the differentially expressed miRNA.

### Identification of biological functions disrupted by dysregulated target genes

To gain insight into biological functions that may be disrupted in ASD as a consequence of altered miRNA expression, the differentially expressed genes whose transcript levels were inversely correlated with those of the differentially expressed miRNAs were uploaded into IPA and Pathway Studio network prediction programs and the target gene networks were generated. For these analyses, a relatively stringent expression level cutoff of log_2_(ratio) ≥± 0.4 was used inasmuch as we are typically able to confirm genes with a log_2_(ratio) ≥± 0.3 by qRT-PCR. Significant biological functions, canonical pathways, and diseases highly represented in the networks were identified using Fisher's exact test (*P *< 0.05).

### Transfection of pre-miRs and anti-miRs

All transfections were performed using siPORT NeoFX Transfection Agent (Applied Biosystems) according to the manufacturer's protocol. Briefly, LCLs were counted and diluted into 2 × 10^5 ^cells/2.3 ml and incubated at 37°C. A total of 5 μl siPORT NeoFX Transfection Agent per transfection condition was diluted and incubated for 10 minutes at room temperature with 95 μl of the prewarmed complete growth media (without antibiotics). Hsa-miR-29b pre-miR precursor, hsa-miR-219b anti-miR inhibitor, Cy3-labeled pre-miR negative control and the Cy3-labeled anti-miR negative control (Applied Biosystems, Foster City, CA, USA) were separately diluted to a final small RNA concentration of 30 nM in 100 μl of complete growth media. Cell suspensions were overlaid onto each of the transfection solutions and mixed gently before incubation at 37°C with 5% CO_2 _for 72 hours. Under these conditions, most cells were observed by fluorescence microscopy to be transfected with Cy3-labeled pre-miR and anti-miR negative controls (Additional file 3), while cytotoxicity, monitored by the MTS cell proliferation assay (Promega, Madison, WI, USA) was determined to be negligible (Additional file 4). Following the 72-hour incubation, the cells were harvested for subsequent analyses.

### Microarray data deposition

All data from the DNA microarray and miRNA microarray analyses have been deposited in the Gene Expression Omnibus (GEO) data repository. The GEO accession number for the miRNA data from this study is [GEO:GSE21086]. The GEO accession numbers for gene expression data for the twin and sib-pair studies are [GEO:GSE4187] and [GEO:GSE15451], respectively.

## Results

### Significantly differentially expressed miRNAs differentiate clinical from non-clinical samples

**Figure 2:**
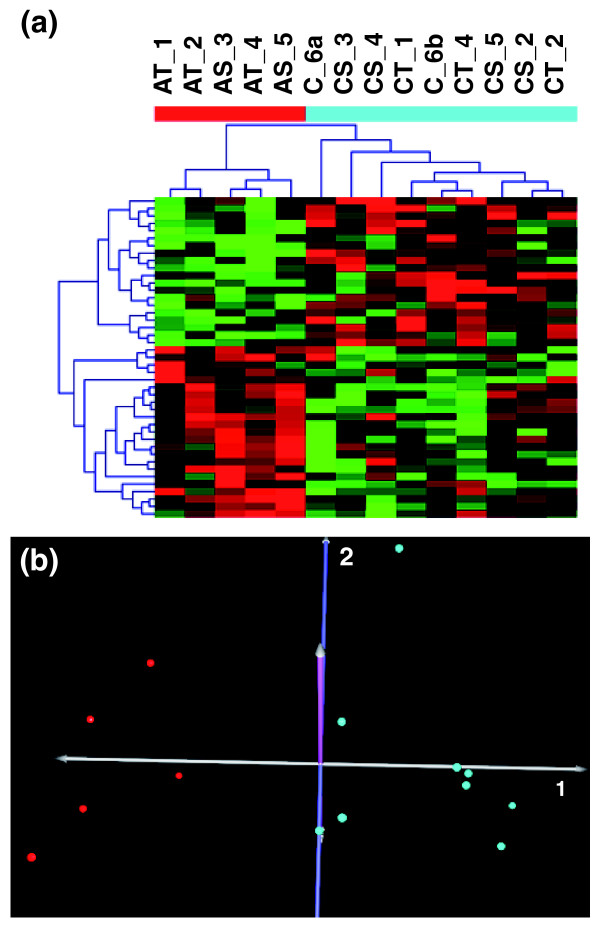
**Hierarchical cluster analysis and principal component analysis of significantly differentially expressed miRNAs from the Pavlidis template matching analysis**. **(a) **Unsupervised hierarchical cluster analysis of 43 significantly differentially expressed miRNAs between all autistic individuals (red bar) and controls (turquoise bar) shows the distinct miRNA expression pattern of the two groups (*P *< 0.05). The individual samples are coded as follows: AT, autistic twin; AS, autistic sibling; CT, control, undiagnosed twin; CS, control, nonautistic sibling; C_6a/b, nonautistic, monozygotic twins a and b. The same numbers following the sample descriptors indicate members of the same family. **(b) **Principal component analysis of the samples based on the same set of miRNAs reduces the dimensionality of the data and shows the clear separation between the autistic individuals (red) and the controls (turquoise).

To identify significantly differentially expressed miRNAs that differentiate clinically discordant individuals, normalized miRNA microarray data were uploaded into the TMeV program for statistical analysis. Pavlidis template matching analysis revealed 43 human miRNAs that were significantly differentially regulated (*P *< 0.05) between autistic and nonautistic individuals. These miRNAs and their corresponding log_2 _ratios for autistic versus control samples are shown in Table 1. Cluster analyses were performed to further determine whether or not the expression levels of these miRNAs could distinguish between the autistic and control groups. Both unsupervised, hierarchical cluster analysis (Figure 2a) and supervised, 2-cluster K-means analysis (data not shown) revealed complete separation of the autistic and control groups based on expression profiles of the differentially expressed miRNAs. Principal component analysis (Figure 2b), which was employed to reduce the dimensionality of the microarray data, also revealed clear separation between autistic individuals and controls based on the 43 significant probes, which was also validated by support vector machine analysis that demonstrated 100% accuracy of class prediction (data not shown).

**Table 1:** Significantly differentially expressed human miRNAs

Clone ID	miRNA	log2 ratio	*P*-value
**Down-regulated**			
SM10801	hsa-miR-182-AS	-1.54	1.44E-03
hSQ018350	hsa-miR-136	-1.50	2.28E-03
SM10637	hsa-miR-518a	-1.45	3.52E-03
hSQ045460	hsa-miR-153-1	-1.41	5.07E-03
SM11115	hsa-miR-520b	-1.38	6.71E-03
SM10529	hsa-miR-455	-1.30	1.25E-02
hHM044864	hsa-miR-326	-1.24	1.95E-02
SM10553	hsa-miR-199b	-1.23	1.96E-02
miR211	hsa-miR-211	-1.23	2.04E-02
hSQ016068	hsa-miR-132	-1.22	2.20E-02
SM10792	hsa-miR-495	-1.20	2.43E-02
hSQ025962	hsa-miR-16-2	-1.19	2.54E-02
hHM044822	hsa-miR-190	-1.18	2.69E-02
hHM044960	hsa-miR-219	-1.17	2.98E-02
hHM045056	hsa-miR-148b	-1.16	3.01E-02
hHM044897	hsa-miR-189	-1.16	3.06E-02
hHM045063	hsa-miR-133b	-1.13	3.59E-02
hSQ018899	hsa-miR-106b	-1.11	4.11E-02
hHM044849	hsa-miR-367	-1.10	4.21E-02
SM10740	hsa-miR-139	-1.10	4.32E-02
			
**Up-regulated**			
hHM044819	hsa-miR-185	1.44	4.04E-03
hHM044919	hsa-miR-103	1.31	1.20E-02
hHM044733	hsa-miR-107	1.26	1.68E-02
hHM044918	hsa-miR-29b	1.24	1.88E-02
hHM045013	hsa-miR-194	1.22	2.11E-02
SM10729	hsa-miR-524	1.22	2.21E-02
hHM044804	hsa-miR-191	1.21	2.23E-02
SM11334	hsa-miR-376a-AS	1.19	2.53E-02
SM10789	hsa-miR-451	1.19	2.64E-02
hHM044971	hsa-miR-23b	1.17	2.95E-02
miR195	hsa-miR-195	1.16	3.02E-02
SM10711	hsa-miR-23b	1.16	3.03E-02
SM10310	hsa-miR-342	1.15	3.24E-02
SM10644	hsa-miR-23a	1.14	3.36E-02
hSQ001775	hsa-miR-186	1.14	3.43E-02
miR25	hsa-miR-25	1.14	3.55E-02
SM10575	hsa-miR-519c	1.13	3.71E-02
SM10238	hsa-miR-346	1.12	3.80E-02
hHM044950	hsa-miR-205	1.12	3.80E-02
hHM044743	hsa-miR-30c	1.11	3.98E-02
hSQ027766	hsa-miR-93	1.10	4.18E-02
hHM045009	hsa-miR-186	1.08	4.67E-02
hHM044831	hsa-miR-106b	1.08	4.86E-02

Forty-three significantly differentially expressed human miRNAs were identified by Pavlidis Template Matching (PTM) analysis (*P *< 0.05). The log_2 _ratios for all miRNAs were calculated from the average of the log_2 _ratio across all autistic samples over the average of the log_2 _ratio across all control samples.

### Biological network prediction of the potential targets revealed a strong association with neurological functions and other biological pathways involved in ASD

Potential target genes for each of the differentially expressed miRNAs were identified using miRBase Targets software [46]. To further identify the biological networks and functions in which these target genes are involved, the target gene list for each miRNA was analyzed using IPA (Table 2). Interestingly, the target genes of 35 out of the 43 human miRNA probes (more than 80% of the significantly differentially expressed miRNAs) were found to be significantly associated with 'neurological functions' or 'nervous system development and function' (Fisher's exact test, *P *< 0.05).

**Table 2:** Ingenuity Pathways Analysis biological functions and pathways associated with potential targets for significantly differentially expressed miRNAs

miRNA	Biological functions/pathways of the miRNA targets (*P*-value) [number of genes]*
hsa-miR-182	**N **(1.18E-03 to 3.86E-02) [59], **E **(1.49E-03 to 3.70E-02) [14]
hsa-mir-136	**G **(1.60E-04 to 3.46E-02) [10], **A **(6.33E-03) [8], **E **(3.50E-03 to 3.46E-02) [21]
hsa-miR-518a	**N **(7.24E-03 to 4.89E-02) [50], **E **(8.57E-05 to 4.44E-02) [20]
hsa-mir-153-1	**N **(1.02E-05 to 2.24E-02) [28], **G **(6.37E-04 to 1.53E-02) [13]
hsa-miR-520b	**N **(2.66E-03 to 4.44E-02) [15],**E **(8.13E-04 to 4.44E-02) [28]
hsa-miR-455	**N **(2.03E-03 to 4.51E-02) [83], **E **(1.06E-03 to 4.51E-02) [42]
hsa-miR-326	**S **(6.24E-04 to 3.99E-02) [28]
hsa-miR-199b	**N **(8.24E-04 to 4.23E-02) [31], **E **(6.04E-03 to 4.23E-02) [21], **S **(5.23E-03 to 4.23E-02) [11]
hsa-miR-211	**N **(7.78E-05 to 2.99E-02) [15],**I **(6.23E-04 to 2.99E-02) [19]
hsa-mir-132	**N **(2.01E-03 to 4.48E-02) [19], **G **(2.01E-03 to 4.48E-02) [23], **E **(2.01E-03 to 4.48E-02) [28]
hsa-miR-495	**N **(6.09E-04 to 4.02E-02) [48], **G **(1.62E-03 to 4.02E-02) [10], **E **(2.51E-04 to 4.02E-02) [24]
hsa-mir-16-2	**N **(8.75E-05 to 4.45E-02) [13], **E **(1.06E-03 to 4.45E-02) [24], **S **(1.58E-03 to 4.45E-02) [17], **Es **(4.86E-02) [9]
hsa-miR-190	**N **(6.63E-04 to 3.86E-02) [39], **G **(2.15E-03 to 3.86E-02) [12], **E **(3.83E-04 to 4.15E-02) [25]
hsa-miR-219	**N **(1.08E-03 to 4.34E-02) [87], **E **(1.88E-03 to 4.34E-02) [11]
hsa-miR-148b	**N **(6.54E-04 to 4.63E-02) [27], **G **(3.81E-04 to 4.63E-02) [27]
hsa-miR-189	**N **(1.57E-03 to 3.76E-02) [23}, **E **(1.57E-03 to 3.76E-02) [19]
hsa-miR-133b	**E **(7.84E-04 to 2.56E-02) [17]
hsa-mir-106b	**N **(1.37E-03 to 4.41E-02) [21], **G **(1.01E-02 to 4.23E-02) [33], **I **(1.54E-03 to 4.38E-02) [18]
hsa-miR-367	**N **(1.35E-03 to 4.37E-02) [20], **G **(1.33E-03 to 4.37E-02) [11]
hsa-miR-139	**G **(1.37E-03 to 4.02E-02) [19], **E **(1.61E-03 to 4.02E-02) [21]
hsa-miR-186	**N **(9.62E-04 to 3.11E-02) [27],**E **(2.83E-03 to 3.11E-02) [14], **S **(9.62E-04 to 3.11E-02) [17], **Es **(1.82E-02) [8]
hsa-mir-93	**N **(2.67E-04 to 4.33E-02) [36], **I **(4.47E-04 to 4.33E-02) [35]
hsa-miR-30c	**N **(9.85E-05 to 4.21E-02) [40], **E **(3.31E-04 to 4.21E-02) [25]
hsa-miR-205	**N **(1.40E-03 to 3.75E-02) [9],**S **(1.19E-04 to 3.75E-02) [23]
hsa-miR-346	**I **(8.61E-04 to 3.03E-02) [56]
hsa-miR-519c	**G **(7.42E-04 to 4.76E-02) [81], **N **(6.58E-03 to 4.71E-02) [25]
hsa-miR-25	**N **(1.04E-04 to 3.61E-02) [39], **Es **(3.95E-02) [8]
hsa-mir-186	**N **(9.62E-04 to 3.11E-02) [27],**E **(2.83E-03 to 3.11E-02) [14], **S **(9.62E-04 to 3.11E-02) [17],**Es **(1.82E-02) [8]
hsa-miR-23a	**N **(1.69E-03 to 4.11E-02) [81], **S **(8.70E-04 to 4.11E-02) [62]
hsa-miR-342	**N **(6.49E-04 to 4.11E-02) [15], **E **(2.13E-03 to 4.11E-02) [12], **S **(6.49E-04 to 4.11E-02) [15]
hsa-miR-23b	**N **(4.31E-05 to 4.01E-02) [87], **S **(3.71E-03 to 4.01E-02) [60], **E **(4.68E-03 to 4.01E-02) [20]
hsa-miR-195	**N **(4.59E-03 to 4.04E-02) [74], **Es **(1.12E-02) [10]
hsa-miR-23b	**N **(4.31E-05 to 4.01E-02) [87], **S **(3.71E-03 to 4.01E-02) [60], **E **(4.68E-03 to 4.01E-02) [20]
hsa-miR-451	**S **(2.99E-04 to 2.43E-02) [29]
hsa-miR-376a	**N **(1.62E-03 to 3.88E-02) [23], **E **(1.62E-03 to 3.10E-02) [10], **S **(1.17E-04 to 4.02E-02) [32], **C **(4.71E-03) [5]
hsa-miR-191	**N **(2.53E-04 to 4.62E-02) [34], **E **(1.87E-03 to 3.93E-02) [12]
hsa-miR-524-3p	**N **(3.44E-04 to 4.47E-02) [66]
hsa-miR-194	**N **(8.47E-03 to 3.86E-02) [24]
hsa-miR-29b	**S **(1.97E-05 to 2.91E-02) [41], **C **(1.63E-03) [6]
hsa-miR-107	**G **(4.81E-04 to 4.13E-02) [46], **E **(1.27E-03 to 4.13E-02), **N **(1.70E-03 to 4.13E-02) [16]
hsa-miR-103	**G **(1.31E-03 to 4.27E-02) [49], **E **(2.01E-04 to 4.27E-02), **S **(3.03E-03 to 4.27E-02) [23], **N **(1.82E-03 to 4.27E-2) [35]
hsa-miR-185	**N **(8.16E-04 to 3.75E-02) [26]

IPA analysis of potential target genes for each of the significantly differentially expressed miRNAs revealed biological functions and pathways associated with the target genes. *P*-values calculated from Fisher's exact test for each function are listed in parenthesis; the number of genes involved in each biological function or pathway is listed in square brackets. The functions are described as: A, androgen and estrogen metabolism; C, circadian rhythm signaling; E, embryonic development; Es, estrogen receptor signaling; G, gastrointestinal diseases/digestive system development and functions; I, inflammatory diseases; N, neurological diseases/nervous system development and functions; S, skeletal and muscular disorders/skeletal and muscular system development and functions.

In addition to gene targets associated with neurological functions, it is noteworthy that a number of the differentially expressed miRNAs also target genes involved in co-morbid disorders associated with ASD, such as muscular and gastrointestinal diseases [50-58]. Target genes of 13 miRNAs (30%) significantly dysregulated in autistic individuals were associated with skeletal and muscular diseases as well as skeletal and muscular development or function. Target genes for 12 significantly dysregulated miRNAs (28%) were associated with gastrointestinal disorders, development, and function, as well as hepatic system disease, hepatic fibrosis, and hepatic cholestasis (*P *< 0.05). It is interesting to note that these disorders are among the most significant biological functions and pathways enriched within the dataset of target genes, inasmuch as ASD individuals are frequently found to have co-morbid diagnoses involving muscle dysfunction (for example, muscular dystrophy, muscle weakness, and hypotonia) and digestive disorders that affect absorption and metabolism.

Another interesting biological function associated with the miRNA gene targets is steroid hormone metabolism. More than 11% (5 out of 43) of the differentially expressed miRNAs showed an association with androgen and estrogen metabolism, as well as with estrogen receptor signaling (*P *< 0.05). Moreover, IPA also showed that target genes for two of the most up-regulated miRNAs - hsa-miR-376a and hsa-miR-29b - were significantly associated with circadian rhythm signaling (Fisher's exact test, *P *= 4.71E-03 and 1.63E-03, respectively).

### Quantitative TaqMan RT-PCR confirmation of selected miRNAs

**Figure 3:**
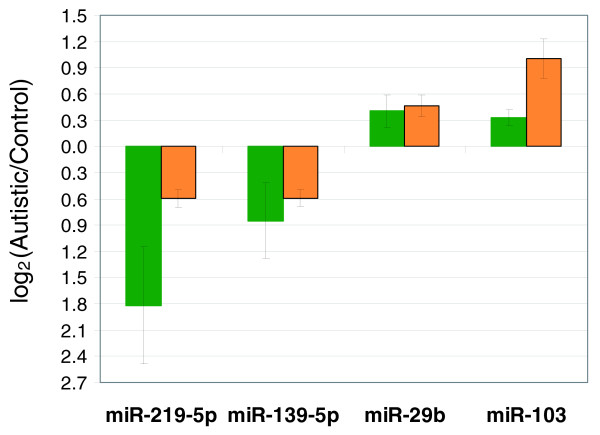
**Results of TaqMan miRNA qRT-PCR analyses of four brain-associated miRNAs (hsa-miR-219-5p, hsa-miR-139-5p, hsa-miR-29b, and hsa-miR-103) in autistic and control lymphoblastoid cell lines**. Expression levels of selected miRNAs associated with brain development from TaqMan qRT-PCR analyses confirm data obtained by miRNA microarrays. Green bars, qRT-PCR data; orange bars, DNA microarray data. Error bars represent standard errors associated with miRNA Taqman qRT-PCR or miRNA microarray analyses (hsa-miR-219-5p/hsa-miR-29b/hsa-miR-103, n = 5 case-control pairs; hsa-miR-139-5p, n = 4 pairs).

MicroRNA TaqMan quantitative RT-PCR (qRT-PCR) analyses were performed to confirm the miRNA expression data of four miRNAs known to be associated with brain development and function. Hsa-miR-29b and hsa-miR-219 are known to be brain-specific, while hsa-miR-139-5p is highly enriched in brain [59-61]. Although not specific to the brain, hsa-miR-103 is highly expressed during corticogenesis [59,62], suggesting an important role in brain development and function. Expression levels of all four brain-associated miRNAs from these analyses were correlated with miRNA microarray data (Figure 3).

### Correspondence between differentially expressed putative target genes and the differentially regulated miRNAs

To examine the possibility that changes in specific miRNAs could result in corresponding changes in the expression levels of the putative target genes, differentially expressed genes from previous cDNA microarray analyses of the same LCLs used in this study [21,40] were compared with the potential target genes of the differentially expressed miRNAs. Of the 3,905 differentially expressed genes between the autistic and control groups, 1,406 (36%) were found to be putative targets of the differentially expressed miRNA, with 1,053 (27%) of these genes exhibiting changes inversely correlated with the respective miRNA changes. These percentages of target genes predicted to be regulated by the miRNA identified in this study are within the range of the approximately 10 to 60% of protein-coding genes that are estimated to be regulated by miRNA [63-65]. Although translational repression is the main mechanism of suppression by miRNA in mammalian cells, the suppressed target mRNA often eventually is degraded in P-bodies [49], thus leading to the expected decreases in transcript levels observed here. A recent study further confirms the effect of miRNA on suppressing target mRNA levels [66].

To increase the stringency of the pathway analyses, an expression level cutoff of log_2_(ratio) ≥± 0.4 was applied to the differentially expressed genes, which reduced the list of potential gene targets to 94 genes. IPA analysis of this set of genes (Table 3) revealed a number of genes significantly involved in neurological disease (*P *= 1.38E-03 to 1.89E-02). Inflammatory diseases, which have also been associated with ASD [22], were found to be significantly associated with the differentially expressed potential target genes (*P *= 2.51E-03 to 2.11E-02). It is interesting to note that lipid metabolism is a cellular function that is a potential target of miRNA regulation. The top canonical pathways implicated by the target genes were nitric oxide signaling (*P *= 1.07E-02), vascular endothelial growth factor (VEGF) signaling (*P *= 1.47E-02), and amyotrophic lateral sclerosis signaling (*P *= 1.88E-02).

**Table 3:** Predicted biological functions from Ingenuity Pathways Analysis

	*P*-value	Number of genes	Genes
**Diseases and disorders**			
Neurological disease	1.38E-03 to 1.89E-02	8	*UCHL1*,*ATF3*,*NDP*,*TUBB2C*,*KIF1B*,*TUBB2A*,*MST1*,*BCL2*
Inflammatory disease	2.51E-03 to 2.11E-02	16	*IL6ST*,*ADM*,*TUBB2C*,*IL32*,*PIK3R1*,*TUBB2A*,*EIF1*,*ALOX5AP*,*MMP10*,*DUSP2*,*BCL2*,*GNAI2*,*HSPA8*,*FUT8*,*LDLR*,*AHNAK*
Skeletal and muscular disorders	2.71E-03 to 1.89E-02	16	*IL6ST*,*ADM*,*COL6A2*,*TUBB2C*,*IL32*,*TUBB2A*,*ALOX5AP*,*MMP10*,*LARGE*,*DUSP2*,*BCL2*,*GNAI2*,*HSPA8*,*CEP290*,*BMI1*,*AHNAK*
**Molecular and cellular functions**			
Lipid metabolism	1.19E-04 to 2.51E-02	13	*ADM*,*IL6ST*,*ABCG5*,*ABHD5*,*IL32*,*PIK3R1*,*ALOX5AP*,*BCL2*,*GNAI2*,*IFRD1*,*LDLR*,*PRKAR2B*,*PITPNC1*
Molecular transport	1.19E-04 to 2.51E-02	12	*IL6ST*,*IFRD1*,*HSPA8*,*GNAI2*,*ABHD5*,*ABCG5*,*LDLR*,*PIK3R1*,*IL32*,*PITPNC1*,*ALOX5AP*,*BCL2*
Small molecule biochemistry	1.19E-04 to 2.51E-02	17	*IL6ST*,*ADM*,*AMPD3*,*ABCG5*,*ABHD5*,*PIK3R1*,*ASS1*,*IL32*,*ALOX5AP*,*BCL2*,*IFRD1*,*GNAI2*,*BCAT1*,*LDLR*,*PITPNC1*,*GOT1*,*GLDC*
Cellular development	1.32E-04 to 2.42E-02	13	*IL6ST*,*ATF3*,*PIK3R1*,*ID3*,*BCL2*,*IGLL1*,*IFRD1*,*ELF3*,*BMI1*,*PRKAR2B*,*PLK2*,*LAMA1*,*PLAC8*
Cell death	2.36E-04 to 1.89E-02	14	*IL6ST*,*ADM*,*ATF3*,*DDIT4*,*PIK3R1*,*NCK1*,*PSIP1*,*SH3BP5*,*ID3*,*BCL2*,*PRKAR2B*,*BMI1*,*PLK2*,*PLAC8*
**Canonical pathways**			
Nitric oxide signaling	1.07E-02	3/90	*CACNA1E*,*PRKAR2B*,*PIK3R1*
VEGF signaling	1.47E-02	3/92	*PIK3R1*,*EIF1*,*BCL2*
Amyotrophic lateral sclerosis signaling	1.88E-02	3/108	*CACNA1E*,*PIK3R1*,*BCL2*
**Toxicity list**			
Hormone receptor regulated cholesterol metabolism	4.96E-02	1/8	*LDLR*

IPA of significant disorders, molecular and cellular functions, canonical pathways, and toxicity genes that are strongly associated with 94 differentially expressed potential target genes of the miRNAs (log_2 _ratio ≥± 0.4). The Fisher's exact *P*-values and the number of genes for each top biological function are listed. VEGF, vascular endothelial growth factor.

### Network prediction of the differentially expressed potential target genes of the differentially expressed miRNAs in ASD

**Figure 4:**
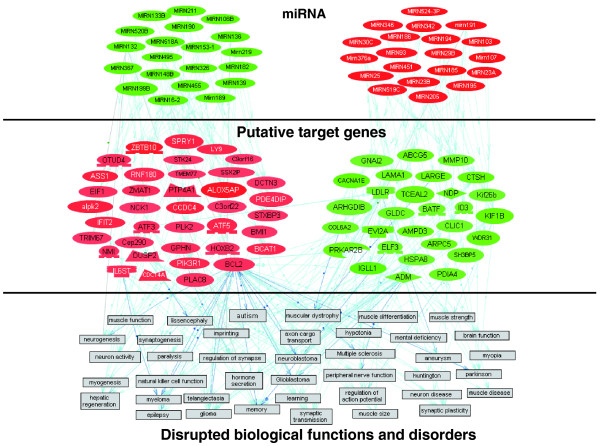
**Relationships between differentially expressed miRNAs, putative target genes, and functions**. Network and pathway analysis using Pathway Studio 5 shows the relationships among the significantly differentially expressed miRNAs, potential target genes (expression cutoff log_2 _ratio ≥± 0.4), and biological functions and disorders implicated by the differentially expressed target genes. Up-regulated genes and miRNAs are in red; down-regulated genes and miRNAs are in green.

The differentially expressed potential miRNA targets were analyzed with Pathway Studio 5 to identify the possible relationships among the target genes and their associated functions (Figure 4). Interestingly, the pathway generated by Pathway Studio revealed relationships between the potential targets of the miRNAs and autism, as well as other neurological functions and disorders previously found to be impacted or associated with ASD, such as memory, regulation of synapses, synaptic plasticity, muscle disease, muscular dystrophy, and muscle strength [50,51,67].

### Validation of miRNA targets

**Figure 5:**
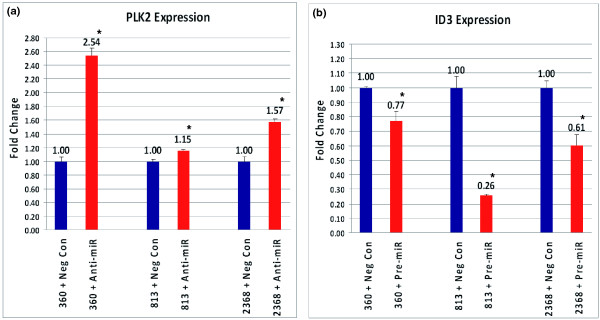
**Validation of miRNA targets**. Three LCLs from non-autistic individuals were transfected with hsa-miR-29b pre-miR precursor, hsa-miR-219b anti-miR inhibitor, pre-miR negative control, or anti-miR negative control. At 72 hours after transfection, qRT-PCR analyses were conducted to determine expression of *PLK2 *and *ID3 *genes in the pre-miR/anti-miR-transfected LCLs (red), compared to respective pre-miR/anti-miR negative controls (navy). **(a, b) **Expression of *PLK2 *was significantly increased in the LCLs transfected with anti-miR-219-5p (a), whereas *ID3 *expression was significantly decreased in pre-miR-29b-transfected LCLs (b). The error bars show the standard error among the technical replicates. *P < 0.05.

Two brain-specific miRNAs (hsa-miR-29b and hsa-miR-219-5p), whose differential expression in ASD was confirmed by TaqMan miRNA qRT-PCR analyses, were selected for miRNA target validation. Among putative target genes of these miRNAs are Inhibitor of DNA binding 3 (*ID3*), which is a target of miR-29b, and Polo-like kinase 2 (*PLK2*), a target of miR-219-5p. ID3 and PLK2 have been associated with circadian rhythm signaling and modulation of synapses, respectively [68-71], and both biological mechanisms have been implicated in ASD [12,14-16,72-79]. To examine whether the overexpression of hsa-miR-29b and the suppression of hsa-miR-219-5p may be responsible for the respective decrease in ID3 and increase in PLK2 transcript levels, LCLs derived from three nonautistic individuals were transfected with hsa-miR-29b pre-miR precursor and hsa-miR-219b anti-miR inhibitor, respectively, to increase hsa-miR-29b and decrease hsa-miR-219-5p activity in the cells. qRT-PCR analyses of the transfected cells revealed the down-regulation of the *ID3 *gene in the LCLs transfected with hsa-miR-29b pre-miR precursor, and the up-regulation of the *PLK2 *gene in the LCLs transfected with hsa-miR-219b anti-miR inhibitor (Figure 5). These results suggest that *ID3 *and *PLK2 *are targets of hsa-miR-29b and hsa-miR-219-5p, respectively. Furthermore, most of the paired comparisons exhibit opposite changes in miRNA and mRNA target expression levels, suggesting that *PLK2 *and *ID3 *are *in vivo *targets of the respective miRNA (Table 4).

**Table 4:** Comparison of miRNA and mRNA expression levels for discordant twins and sib pairs for miR-219 and its target, *PLK2*, and for miR-29b and its target, *ID3*

miRNA (target)	A361/C360	A809/C810	A809/C813	A2369/C2368	A2369/C2357	A366/C365	A2769/C2772	Average
miR-219 (*PLK2*)	-1.447 (0.414)	-1.089 (0.147)	-2.330 (NA)	-2.390 (NA)	-0.175 (NA)	0.398 (0.314)	-1.176 (0.456)	-1.173 (0.333)
miR-29b (*ID3*)	0.585 (-0.406)	1.720 (-0.187)	1.287 (-0.574)	0.395 (-0.603)	1.315 (0.070)	2.939 (-0.152)	0.061 (-0.233)	1.186 (-0.298)

The first three columns are log2 ratios for discordant monozygotic twins, while the last four columns are ratios for sib-pair comparisons. NA, no expression ratio obtained for this gene because an intensity value for either the autistic or control sample was missing.

## Discussion

### miRNA expression in autism spectrum disorders

In this study, we demonstrate the differential expression of 43 miRNA species in LCLs from individuals with ASD relative to controls (Table 1), 16 of which are brain-specific, brain-related, or involved in neural differentiation [59-62]. Although the total number of samples in this study is modest, the use of discordant monozygotic twins and sibling case-controls offers the ability to identify differences in miRNA against the same or closely related genotype, which is an advantage in investigations of epigenetic mechanisms contributing to autism. We have previously used this strategy in first identifying gene expression differences in these same monozygotic twins [21] and sibling case-controls [40], and then validated our initial findings with a larger study involving 116 unrelated case-controls [77]. Here, we further utilize the original gene expression data of these same samples to demonstrate that differentially expressed miRNA can account for approximately 36% of the differentially expressed transcripts [21,40], thus implicating miRNA as a potent regulator of gene expression in ASD. Functional analyses of the putative gene targets that show inverse correlation with the expression of miRNA reveal numerous processes relevant to or associated with ASD that are potentially regulated by the differentially expressed miRNA (Table 2, Figure 4). These processes include embryonic development, synaptic development and function, circadian rhythm signaling, inflammation, androgen metabolism, and digestive functions, mirroring the major findings of our gene expression analyses [21,40,77] Significantly, we verify inverse changes in the levels of putative target genes of two of the altered brain-specific miRNAs through the use of anti-miRs (for knockdown) and pre-miRs (for overexpression) (Figure 5).

To date, only two other studies have conducted miRNA expression profiling of autistic individuals. Talebizadeh and colleagues [38] evaluated the global expression of 470 known human miRNAs using LCLs derived from six autistic individuals and six sex- and age-matched controls by miRNA microarray assays. Of these 470 miRNAs, they found nine that were significantly differentially expressed in the autistic samples. Three of the nine miRNAs were replicated in our study, with similar up-regulation of miR-23a and miR-23b, but down-regulation of miR-132. Although we have no specific explanation for this contrasting result for miR-132, differences between our study and that of Talebizadeh *et al*. [38] include our use of related samples (that is, co-twins/siblings) as controls, a custom-printed rather than commercial platform, and the restriction of our study to male subjects. Additional analyses are thus required to further explain the differences in miRNA expression data between these two studies on LCLs.

Abu-Elneel *et al*. [39] investigated the expression of 466 human miRNAs in postmortem cerebellar cortex tissue of 13 autistic individuals using multiplex quantitative PCR and found 13 down-regulated and 16 up-regulated miRNAs. Interestingly, the up-regulation of miR-23a and down-regulation of miR-106b reported in the autistic cerebellar cortex were also found in our study using LCLs. Predicted potential target genes of miR-23a were found to be associated with neurological diseases and skeletal and muscular system development and functions, whereas those of miR-106b were associated with neurological diseases, inflammatory diseases, and gastrointestinal diseases (Table 2). These findings support the hypothesis that miRNA dysregulation in peripheral blood cells can reflect at least some miRNA alterations occurring in the brain, thus lending support to the use of LCLs as a surrogate tissue to study miRNA expression in individuals with ASD.

### Brain-related miRNAs are differentially expressed in LCLs from ASD patients

Our earlier studies profiling gene expression in LCLs from monozygotic twins and siblings discordant for diagnosis of autism and unrelated autistic case-controls reveal the differential expression of hundreds to thousands of genes [21,40,77], suggesting that higher level epigenetic gene regulatory mechanisms are involved in ASD. The present study provides further insight into the post-transcriptional gene regulatory network associated with ASD by identifying differential miRNA expression as one mechanism for the differential gene expression associated with ASD. Interestingly, at least 16 of these miRNAs have been previously reported by Sempere and colleagues [59] to be brain-specific, brain-enriched, or induced by neuronal differentiation. Krichevsky and colleaques [62] reported significant changes in the expression of nine miRNAs during brain development; one of these miRNAs (miR-103) was also significantly differentially expressed in our study. Thus, the differential expression of these brain-related miRNAs in LCLs suggests that gene expression differences previously observed in LCLs [21,40,77] may reflect similar changes in the brain, possibly due to global or system-wide dysregulation of miRNA expression.

### Biological functions associated with the confirmed miRNAs and their target genes

Using miRNA TaqMan qRT-PCR, we confirmed four differentially expressed miRNAs (hsa-miR-219-5p, hsa-miR-139-5p, hsa-miR-29b, and hsa-miR-103) previously reported to be associated with the brain [59-62]. Of the confirmed miRNAs, we observed a significant decrease in brain-specific hsa-miR-219, which is associated with circadian rhythm and *N*-methyl-*D*-aspartate (NMDA) glutamate receptor signaling, both of which have been implicated in ASD [72-77,80,81]. In particular, Kocerha and colleagues [82] found that disruption of NMDA receptor signaling resulted in decreased levels of miR-219 in mice. Hypofunction of NMDA receptor signaling has been associated with a number of neurological disorders, including autism [83-85], attention deficit hyperactivity disorder [86,87], and schizophrenia [88]. One of the putative target genes whose expression was confirmed to be inversely correlated with hsa-miR-219 expression is *PLK2 *(Figure 4), a serine/threonine kinase expressed in the brain [89] that participates in regulation of cell cycle progression [90] and homeostatic plasticity of hippocampal neurons [69,70]. A recent study found that PLK2 was induced during prolonged epileptiform activity, and was required for the activity-dependent reduction in membrane excitability of pyramidal neurons, suggesting PLK2's role in preventing escalating potentiation and in maintaining synapses in a plastic state [71]. PLK2 induction in hippocampal neurons resulted in weakening of synapses through phosphorylation and degradation of post-synaptic spine-associated Rap GTPase-activating protein (SPAR), a regulator of actin dynamics and dendritic spine morphology [69,71], leading to loss of mature dendritic spines and synapses [91,92]. Over-expression of PLK2 in individuals with ASD due to decreased hsa-miR-219 levels as observed in this study (Figure 5, Table 4) may thus lead to global reduction in synaptic strength and neuronal excitability, which could be partially responsible for the synaptic dysfunction implicated in ASD.

Another confirmed brain-specific miRNA differentially expressed in individuals with ASD is hsa-miR-29b. Besides its confirmed target, *ID3 *(Figure 5), which is involved in regulating the biological clock (see below), other target genes that show expression levels inversely correlated with the over-expression of this miRNA include *COL6A2 *(Collagen, type VI, alpha 2), *CLIC1 *(Chloride intracellular channel 1), *ARPC5 *(Actin related protein 2/3 complex, subunit 5, 16 kDa), and *KIF26b *(Kinesin family member 26B). Interestingly, a number of mutations in *COL6A2 *have been observed in muscular disorders, including Bethlem myopathy [93-95] and Ullrich congenital muscular dystrophy [94,96-98]. Mutation in the *COL6A2 *gene results in decreased *COL6A2 *transcript, leading to disruption of collagen formation and stability, which results in decreased muscle strength [93]. A number of motor impairments and muscular disorders, including muscular dystrophy, hypotonia, and muscle weakness, are observed in individuals with ASD [50,99,100]. It is therefore interesting to postulate that suppression of *COL6A2 *as a result of up-regulated hsa-miR-29b may be one of the genetic mechanisms underlying muscular disorders and motor impairments frequently observed in individuals with ASD.

Among brain-enriched miRNAs [59], hsa-miR-139-5p was selected for confirmation analysis using miRNA TaqMan qRT-PCR assay. Although the precise targets in brain are not known, one of its putative targets (myomegalin or *PDE4DIP *(Phosphodiesterase 4D interacting protein)) is a homolog of brain-enriched *CDK5RAP2 *(CDK5 regulatory subunit associated protein 2), a gene that regulates brain size [101-104], which has been shown to be abnormal in ASD [105-119]. Interestingly, this miRNA has been shown to be involved in prion-induced neurodegeneration [120].

Two of the most up-regulated miRNAs, miR-103 and miR-107 (Table 1), have been reported to be paralogous miRNAs. miR-103 and miR-107 are expressed in many human organs, with the highest concentrations occurring in brain tissue [121]. Furthermore, miR-103 was demonstrated to change during corticogenesis in mice [62]. Although the specific targets of miR-103/107 in brain are unknown, these miRNAs are known to be associated with lipid metabolism [121], and in fact reside within introns of the pantothenate kinase (*PANK*) genes, which catalyze the biosynthesis of Coenzyme A, a critical component in fatty acid biosynthesis and oxidation. It should be noted that, while *PANK *was not found to be among the significantly differentially expressed genes in this study, it was found to be increased in ASD and in the same direction as miR-103/107 in our previous study of a larger cohort of 31 autistic individuals with severe language impairment and 29 controls [77]. Aside from the association of *PANK *mutations and a neurodegenerative (Hallervorden-Spatz) disease [122,123], alterations in lipid and fatty acid metabolism are also known to be associated with ASD. Vancassel and colleagues [124] examined the levels of phospholipid fatty acids in the plasma of individuals with ASD compared to controls with mental retardation and found significant reductions in docosahexaenoic acid (22:6n-3) levels in autistic individuals, resulting in significantly lower levels of total n-3 polyunsaturated fatty acids. The dysregulation of miR-103/7 may therefore contribute to abnormal lipid and fatty acid metabolism in ASD.

### miRNAs regulating circadian rhythm are significantly dysregulated in ASD

Recently, dysregulation of circadian rhythm has been considered as a mechanism for impairments in neurological and other functions (for example, sleep, digestive) in ASD [72-77]. In particular, the circadian rhythm (or 'clock') genes have been posited to underlie social timing deficits associated with autism [72], as well as lead to the sleep disorders frequently observed in ASD [125,126]. Bourgeron [75] also proposed an important role for circadian rhythm with respect to regulation of synaptic genes (*NLGN3 *(Neuroligin 3), *NLGN4 *(Neuroligin 4), *NRXN1 *(Neurexin 1), and *SHANK3 *(SH3 and multiple ankyrin repeat domains 3)), thus affecting susceptibility to ASD. Our large-scale genomic study also found strong support for an association between ASD and circadian rhythm dysfunction [77]. Interestingly, as many as 15 circadian rhythm genes, including *AANAT *(Arylalkylamine-N-acetyltransferase), *BHLBH2 *(Class B basic helix-loop-helix protein 2), *CRY1 *(Cryptochrome 1 (photolyase-like)), *NPAS2 *(Neuronal PAS domain protein 2), *PER1 *(Period homolog 1), *PER3 *(Period homolog 3), and *DPYD *(Dihydropyrimidine dehydrogenase), were differentially expressed exclusively in the most severe phenotype of ASD, which was characterized by severe language impairment [77,127]. It is interesting to note that two of the most significantly down-regulated miRNAs (miR-219 and miR-132) in individuals with ASD have been reported to be involved in modulating the master circadian clock located in the suprachiasmatic nucleus [128-131]. Specifically, brain-specific miR-219 was a target of the master circadian regulator CLOCK and BMAL1 (Brain and muscle ARNT-like 1) complex, exhibited robust circadian rhythm expression, and fine-tuned the length of the circadian period in mice [130,131]. It is relevant, therefore, that we demonstrate that *PLK2*, which is involved in circadian rhythm signaling, is a target of miR-219 (Figure 5).

Functional analyses of putative target genes using IPA (Table 2) also showed that other miRNAs (hsa-miR-29b and hsa-miR-376a) are significantly associated with circadian rhythm signaling, with hsa-miR-29b targeting the *ID3 *gene, which might be important for entrainment and operation of the mammalian circadian system through ID3 interaction with CLOCK and BMAL1 [68]. Significantly, we show that hsa-miR-29b pre-miR precursor results in the down-regulation of *ID3 *transcript. *ID3 *is also a neuronal target of *MeCP2 *(Methyl CpG binding protein 2), which is the causative gene for Rett syndrome [132]. Other putative targets of brain-specific hsa-miR-29b are genes known to interact in the regulation of the biological clock, including *ARNTL *(Aryl hydrocarbon receptor nuclear translocator-like; *BMAL1*), *ATF2 *(Activating transcription factor 2), *DUSP2 *(Dual specificity phosphatase 2), *PER1*, *PER3*, and *VIP *(Vasoactive intestinal peptide). Although only *DUSP2 *was found to be differentially expressed in the current analysis, it is interesting to note that our recent large-scale gene expression study of LCLs from over 100 unrelated case-controls found significant decreases in *PER1 *and *PER3 *transcript levels in individuals with the most severe phenotype of ASD [77]. However, further experimental studies are required to determine whether or not the over-expression of hsa-miR-29b results in the suppression of these two *PER *genes.

### Target genes of miRNAs involved in functions and processes associated with ASD

To obtain more insight into the biological functions regulated by each of the differentially expressed miRNAs, the potential target genes of each miRNA were predicted *in silico *and uploaded into IPA network prediction software. For most miRNAs, target genes were predicted to be involved in neurological disease and nervous system development and function on the basis of gene enrichment within the dataset (Table 2). This finding suggests that the significantly differentially expressed miRNAs may lead to post-transcriptional dysregulation of target genes that, in turn, leads to the disruption in neurological functions contributing to ASD pathophysiology.

The dysregulation of these specific miRNAs may also potentially impact other physiological functions. Besides the neurological functions, almost half of the differentially expressed miRNAs targeted a number of genes involved in gastrointestinal disorders and hepatic diseases, which have been found in approximately 50% of individuals with ASD [133,134]. Our findings thus provide a plausible explanation for some of the systemic effects observed in ASD that affect other organs in addition to the nervous system.

Steroid hormones have been suggested to be involved in the etiology or susceptibility to ASD [135,136]. In particular, previous studies have reported elevated androgen levels in the serum of autistic individuals, including females [135,136], and we have recently reported changes in genes in LCLs that correlated with increases in testosterone [40,77]. Androgens and estrogens are known to participate in synaptic plasticity in the brain of rats. Whereas estrogens have been found to take part in synaptic plasticity in the hippocampus of female rats [137], androgens can modulate that function in both male and female rats [138]. Within this context, it is noteworthy that four of the differentially expressed miRNAs (miR-16, miR-186, miR-25, and miR-195) target genes participating in estrogen receptor signaling. miR-136, which was one of the most down-regulated miRNAs found among all five ASD samples, is also associated with androgen and estrogen metabolism.

miRNAs are known to act through translational repression [23-27]. However, the repressed transcripts are often degraded in P-bodies, ultimately leading to reduced transcript levels for a particular miRNA-repressed gene [49]. This inverse correlation between miRNA and target gene transcript levels is further suggested by the observed inverse correlation between miRNA 'host' genes and the miRNA target transcripts using a novel analysis called HOCTAR (for 'host gene oppositely correlated targets') [66]. Thus, an increase in a particular miRNA is likely to lead to decreased transcript levels of target genes and *vice versa*. However, inverse correlation of miRNA and target mRNA levels is not necessarily observed. Nevertheless, comparing the miRNA expression data obtained by the present study with data obtained by our previous cDNA microarray analysis of these same samples reveals that the direction of change for roughly 27% of the differentially expressed genes was inversely correlated with that of the respective potentially regulatory miRNAs. Relational gene networks constructed using computational network prediction tools show that the inversely correlated target genes of the significantly differentially expressed miRNAs are linked to autism as well as to co-morbid disorders frequently reported in many autistic individuals (Figure 3). For example, a number of genes in the network are linked to synaptic function, such as regulation of synapse, synaptic plasticity, and synaptic transmission. Synaptic plasticity has been comprehensively described in the context of fragile X syndrome and linked to autism [139]. FMRP (Fragile X mental retardation protein), the key protein missing in fragile X syndrome, is an RNA binding and transport protein that regulates the translation of many other proteins important for synaptic plasticity, including neuroligins 3 and 4 and SHANK, all of which have been previously associated with autism [12,13,139,140] Muscular dystrophy and muscle disease are also known to be among the co-morbid disorders frequently found in autism [99]. Thus, putative target genes of the differentially expressed miRNAs identified in this study can be associated with both neurological as well as co-morbid features of ASD.

Although the major behavioral symptoms of ASD appear to be of neurological origin, the prevalence of gastrointestinal abnormalities, hypotonia, and immune disorders in individuals with ASD have led some researchers to view ASD more as a systems disorder that is a result of gene and environment interactions. Thus, several recent studies, including three from our laboratory [21,40,77], have used LCLs as a surrogate experimental model to better understand the pathobiology of ASD as well as to identify peripheral biomarkers of ASD for diagnostic purposes [21,38,40,77,127,141,142]. In particular, our previous study of monozygotic twins discordant for diagnosis or severity of autism revealed differentially expressed genes with known neurological functions of potential relevance to autism [21]. Because identical twins share the same genotype, this study suggested the involvement of epigenetic factors in the regulation of gene expression in ASD. Furthermore, the global scale of the observed changes in gene expression suggested the operation of 'master switches' that can activate or suppress multiple genes at once. Non-coding RNAs, including miRNAs, are potential epigenetic regulators of gene expression and can operate in this fashion [24,143-146].

## Conclusions

Our miRNA expression profiling study of LCLs derived from individuals with ASD, their discordant monozygotic co-twins, and/or their unaffected siblings reveals a set of significantly differentially expressed miRNAs whose target genes are associated with neurological diseases and functions. Moreover, by integrating and correlating both miRNA and gene expression data from the same samples, we take a systems biology approach to reducing the total number of relevant targets for further study as candidate ASD genes. Finally, the significant differential expression of brain-specific and brain-related miRNAs detected in LCLs may reflect systemic changes underpinning ASD that give rise to neuropathological conditions and, moreover, support the use of LCLs as a surrogate tissue to study miRNA expression in ASD.

## Abbreviations

AGRE: Autism Genetic Resource Exchange; ASD: autism spectrum disorders; GEO: Gene Expression Omnibus; IL: interleukin; IPA: Ingenuity Pathway Analysis; LCL: lymphoblastoid cell line; miRNA: microRNA; NMDA: *N*-methyl-*D*-aspartic acid; *P*-org: *P*-orthologous; qRT-PCR: quantitative reverse-transcription PCR; TMeV: TIGR Multiexperiment Viewer; UTR: untranslated region.

## Competing interests

The authors declare that they have no competing interests.

## Authors' contributions

TS performed all of the experiments and wrote the manuscript for this study. RZ and GC trained TS in miRNA analysis in the laboratory of HKM who also provided material support for the miRNA microarray analyses. VWH conceived of and designed the study, and also participated in the writing of this manuscript.

## Supplementary Material

Additional file 1 **Complete list of non-coding RNA probes on custom microarray printed by NIH Microarray CORE Facility**. The first sheet in the Excel workbook describes all the non-coding RNAs on the array. The second sheet in the Excel workbook identifies the human miRNAs that were considered in this study. Click here for file

Additional file 2 **List of 3,905 differentially expressed genes between discordant twins and between sib pairs after meta-analysis of combined gene expression data**. Differential expression is expressed as log_2 _ratio of expression between the autistic individual and his undiagnosed or unaffected twin/sibling. Click here for file

Additional file 3 **Assessment of transfection efficiency of pre-miRs and anti-miRs**. LCLs from non-autistic individuals were transfected with **(a) **Cy3-labeled pre-miR negative control and **(b) **Cy3-labeled anti-miR negative control. Most of the cells appear fluorescent, indicating uptake of the pre-miR and anti-miR into the cells. Click here for file

Additional file 4 **Cytotoxicity assays for transfection of pre-miRs and anti-miRs**. MTS cell proliferation assays (Promega) were conducted to determine the number of viable cells in three nonautistic LCLs after transfection with **(a) **30 nM pre-miRs, or **(b) **30 nM anti-miRs, for 72 hours. No significant cytotoxicity was found under any transfection condition. Click here for file
