# Supplementary figures and images for: Investigation of post-transcriptional gene regulatory networks associated with autism spectrum disorders by microRNA expression profiling of lymphoblastoid cell lines
Source: Genome Med. 2010 Apr 7;2(4):23. doi: 10.1186/gm144 (PMC2873801; doi:10.1186/gm144)

## Slide 1
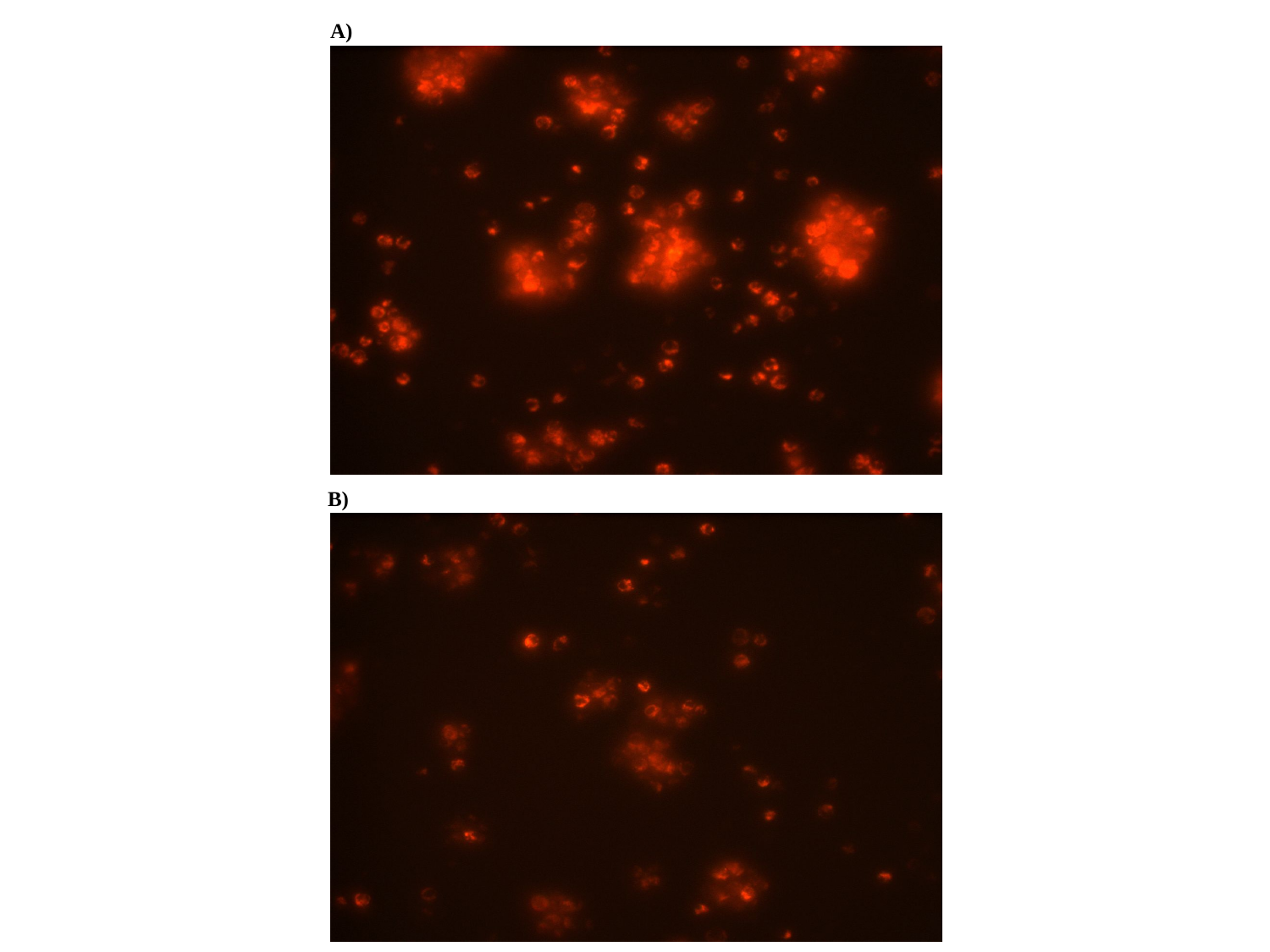

A)
B)

Supplement: Additional file 3 — Assessment of transfection efficiency of pre-miRs and anti-miRs. LCLs from non-autistic individuals were transfected with (a) Cy3-labeled pre-miR negative control and (b) Cy3-labeled anti-miR negative control. Most of the cells appear fluorescent, indicating uptake of the pre-miR and anti-miR into the cells. [file gm144-S3.PPT]

## Slide 1
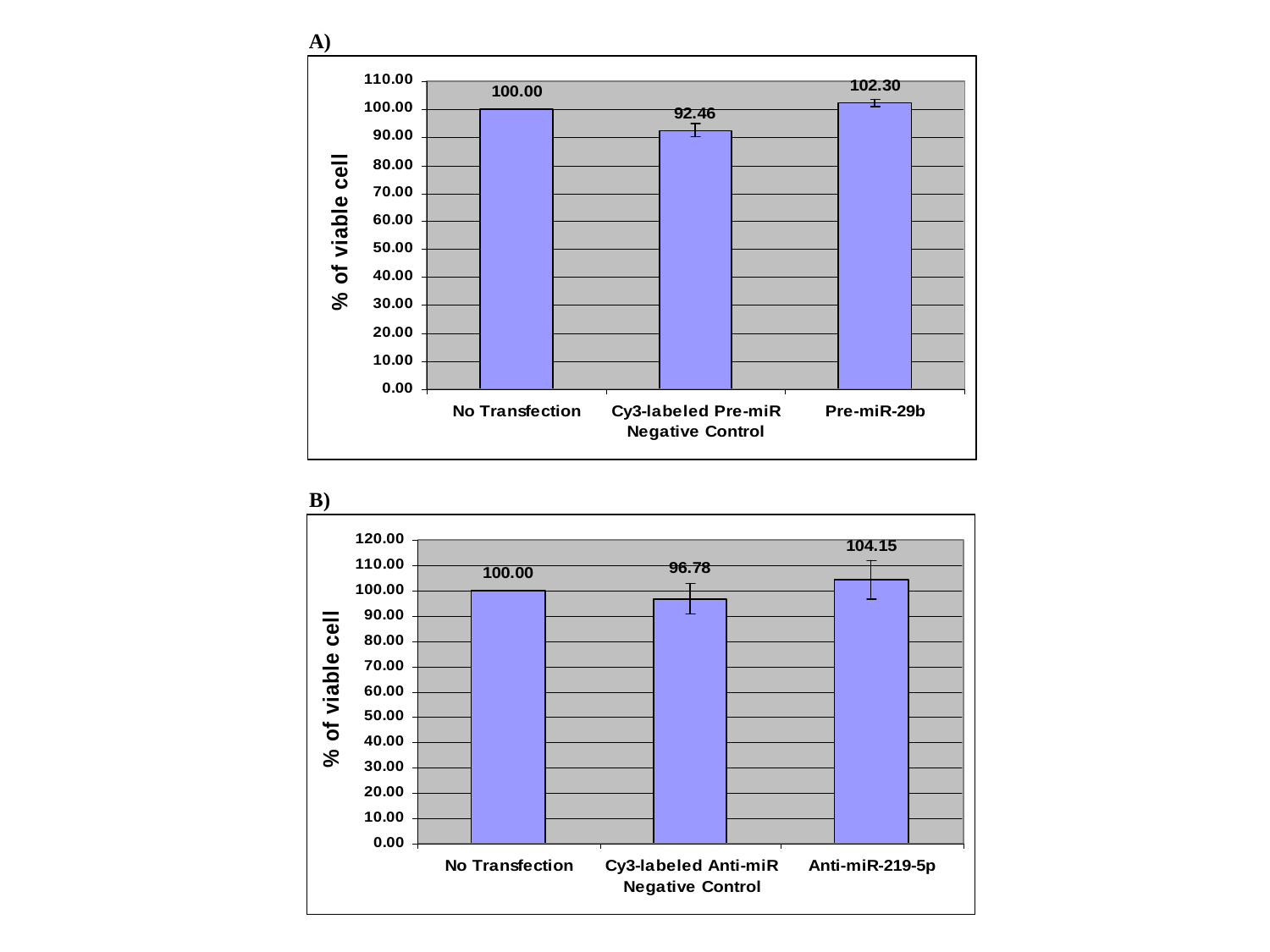

A)
B)

Supplement: Additional file 4 — Cytotoxicity assays for transfection of pre-miRs and anti-miRs. MTS cell proliferation assays (Promega) were conducted to determine the number of viable cells in three nonautistic LCLs after transfection with (a) 30 nM pre-miRs, or (b) 30 nM anti-miRs, for 72 hours. No significant cytotoxicity was found under any transfection condition. [file gm144-S4.PPT]
